# Characterization bacterial metabolites and peripheral immune cell populations in stable and progressive Alzheimer's disease

**DOI:** 10.1002/alz70855_107794

**Published:** 2025-12-24

**Authors:** Ethan B Loew

**Affiliations:** ^1^ University of Massachusetts Chan Medical School, Worcester, MA, USA

## Abstract

**Background:**

Alzheimer's disease (AD) is the most common type of dementia which results in debilitating memory loss as the disease advances. However, among older adults with AD, some may experience rapid cognitive decline while others may maintain a stable cognitive status for years. In addition to the amyloid plaques, tau tangles, and neuronal inflammation characteristic of AD, there is strong evidence of dysregulation in the peripheral immune system, including decreased naïve T cells and increased memory T cells among older adults with AD. It is currently unknown what underlies dysfunction in the peripheral immune system or whether changes in peripheral immune cells are associated with cognitive decline.

**Method:**

We have performed unbiased stool metabolomics combined with machine leaning to identify bacterial metabolites associated with AD versus propensity matched healthy controls. In our ongoing work, we are longitudinally characterizing resting peripheral immune cell populations by flow cytometry and gut microbiome composition by metagenomic sequencing.

**Result:**

We have identified an increase in the metabolites methionine sulfone, homocysteine, and cysteine in the stool of older adults with AD compared to controls and found machine learning models supported bacterial methionine production as a key AD associated variable. Among the population of AD patients experiencing cognitive decline, determined by increasing ADAS‐Cog score >6 points over one year (*n* = 10 declining vs *n* = 8 stable cognition), we have identified increases in the bacterial genes responsible for methionine production at the point of cognitive decline compared to previous timepoints and between patients with decline versus stable cognition. In accordance with the role of methionine in promoting immune cell proliferation and differentiation, we have compared the composition of peripheral immune cells among adults with declining versus stable cognition and identified increased CD4^+^ effector memory T cells at the point of cognitive decline.

**Conclusion:**

This longitudinal clinical study identifies changes in stool metabolites and resting peripheral T cell populations in AD patients and among AD patients with cognitive decline. We propose that gut bacterial produced methionine acts to promote peripheral immune differentiation and dysfunction, leading to cognitive decline in AD.

